# Supplementary material for: The Global Influence of Sodium on Cyanobacteria in Resuscitation from Nitrogen Starvation
Source: Biology (Basel). 2023 Jan 19;12(2):159. doi: 10.3390/biology12020159 (PMC9952445; doi:10.3390/biology12020159)
Supplement: Supplementary file 1 [file biology-12-00159-s001.zip › Legends_Supplementary.pdf]

- Supplementary Figure S1: minimal sodium concentration required for vegetative growth. A depicts cells grown in 24 well plates for 7 days in increasing sodium concentrations. CL =continuous light, D/N = day night cycle, gas exchange = higher gas exchange enabled thorugh shaking, low exchange = plates were left standing, limiting gas exchange. B depicts the OD_{750} as indicator of cell mass for cells grown in 100, 200 or 500 µM NaCl for 7 days. Cells were either cultivated shaking or standing, in continuous light or in a 12 hour day/ 12 hour night cycle. Each data-point represents measured triplicates. Error bars represent the SD.

Supp. Fig. 2: extracellular concentration of nitrogen compounds in recovery. Recovery was initiated by addition of either 1 mM of KNO_3 or NH_4Cl. Cells were recovered either in absence of sodium or in addition of 1 mM NaCl. Y-axis depicts the unit of concentration and the measured compound, x-axis the time after addition of nitrogen source in hours. A shows the amount of nitrate, B of nitrite and C of ammonium measured. Each data-point represents measured triplicates. Error bars represent the SD.
