# Supplementary figures and images for: The Global Influence of Sodium on Cyanobacteria in Resuscitation from Nitrogen Starvation
Source: Biology (Basel). 2023 Jan 19;12(2):159. doi: 10.3390/biology12020159 (PMC9952445; doi:10.3390/biology12020159)

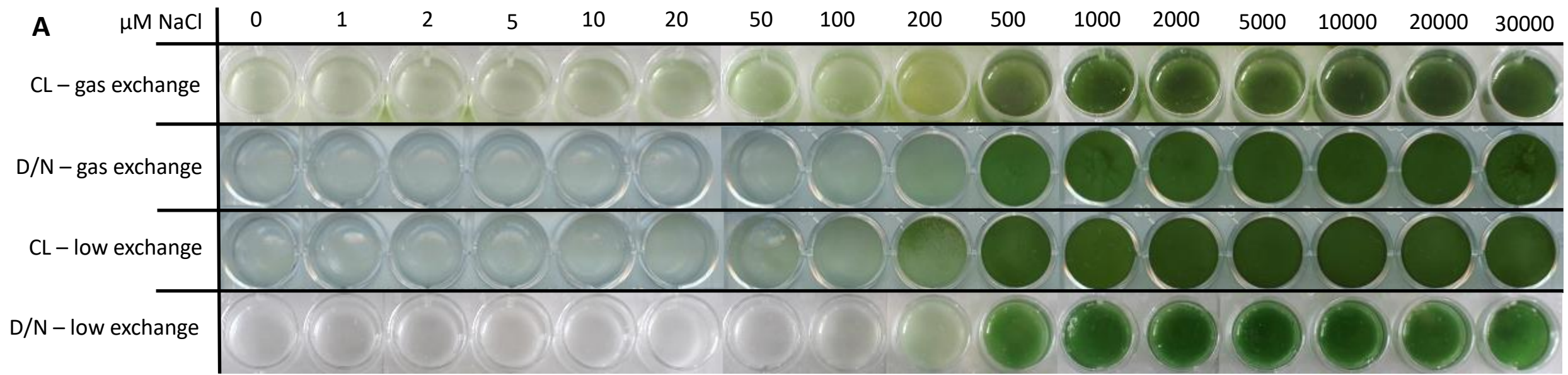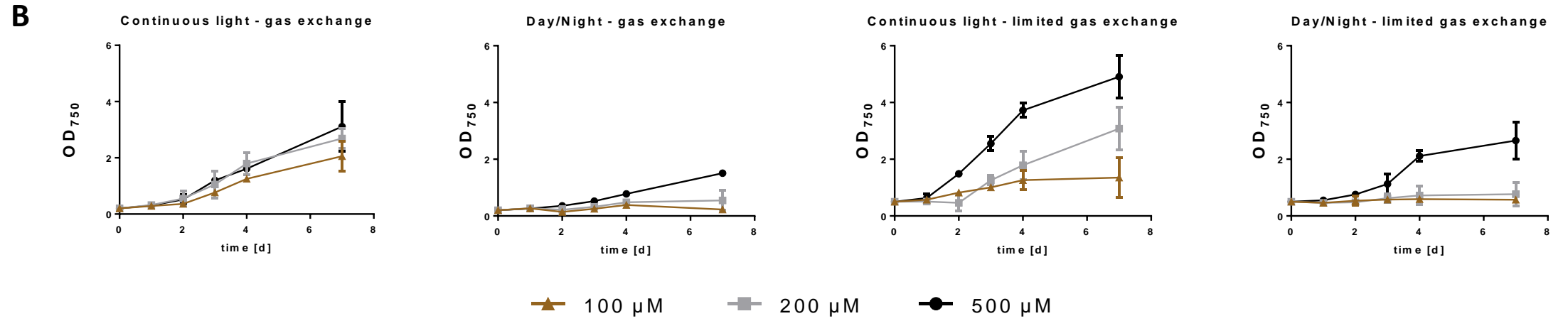

Supplement: Supplementary file 1 [file biology-12-00159-s001.zip › Figure_S1.pdf]

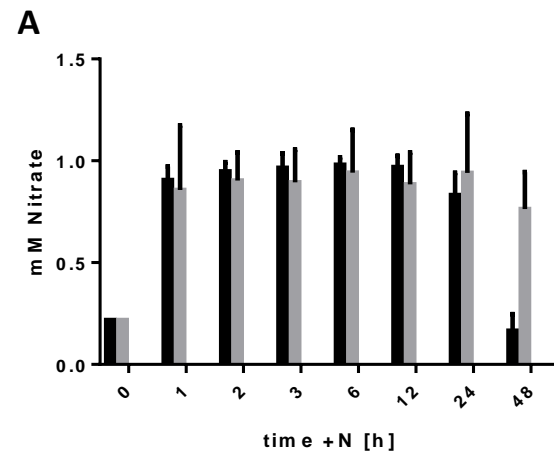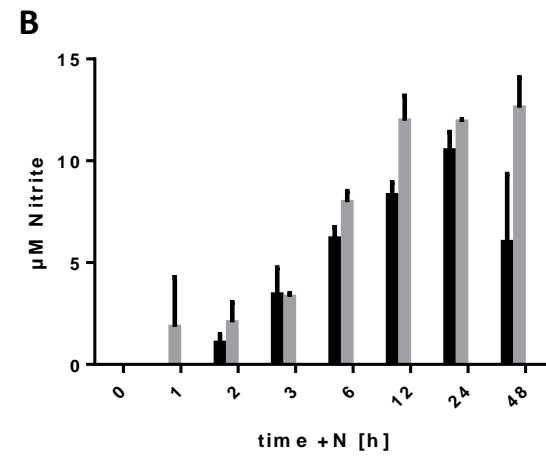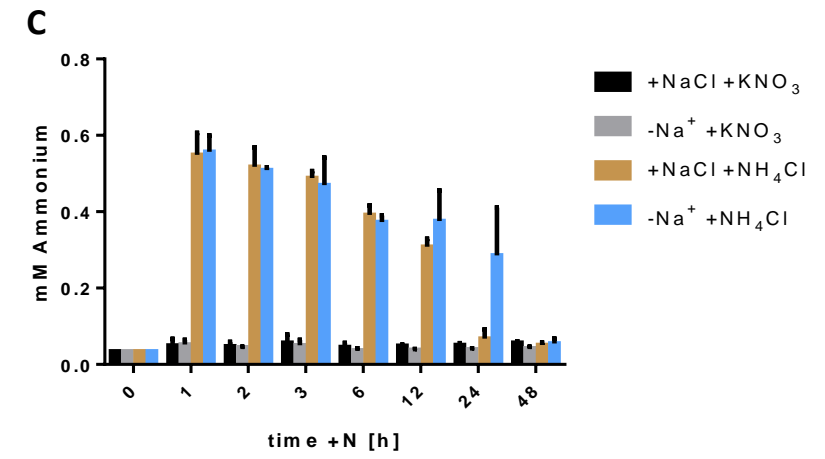

Supplement: Supplementary file 1 [file biology-12-00159-s001.zip › Figure_S2.pdf]
